# Interannual Fluctuations in Mean Straight Carapace Length (SCL) of Nesting Kemp’s Ridley Sea Turtles Signal Demographic Shifts at Rancho Nuevo Sanctuary, Tamaulipas, Mexico

**DOI:** 10.3390/life16040631

**Published:** 2026-04-08

**Authors:** Kevin A. Zavala-Félix, Fátima Yedith Camacho-Sánchez, Valeria Leal-Sepúlveda, Héctor Hugo Acosta-Sánchez, A. Alonso Aguirre, Alan A. Zavala-Norzagaray, Catherine E. Hart, César P. Ley-Quiñónez, Miguel Angel Reyes-López

**Affiliations:** 1 Instituto Politécnico Nacional (IPN-CIIDIR Sinaloa), Guasave 81049, Sinaloa, Mexico; kevinzf26@gmail.com (K.A.Z.-F.); valeriasepulveda2116@gmail.com (V.L.-S.); anorzaga@ipn.mx (A.A.Z.-N.); 2 Universidad Autónoma de Tamaulipas, UAM Reynosa-Aztlan, Reynosa 88740, Tamaulipas, Mexico; fatimaycs@gmail.com; 3 Laboratory of Conservation Medicine, Centro de Biotecnología Genómica, Instituto Politécnico Nacional, Reynosa 88700, Tamaulipas, Mexico; 4 United Nations Development Programme, Comisión Nacional de Áreas Naturales Protegidas, Aldama 89678, Tamaulipas, Mexico; hectorhugo8486@gmail.com; 5 Department of Fish, Wildlife, and Conservation Biology, Warner College of Natural Resources, Colorado State University, Fort Collins, CO 80523, USA; a.alonso.aguirre@colostate.edu; 6 Uella Environmental Research and Consulting, Bahía de Banderas 63732, Nayarit, Mexico; cehart03@gmail.com

**Keywords:** *Lepidochelys kempii*, Kemp’s ridley, straight carapace length, nesting females, demographic monitoring, Rancho Nuevo, Tamaulipas, Mexico, Deepwater Horizon, population recovery

## Abstract

The critically endangered Kemp’s ridley sea turtle (*Lepidochelys kempii*) population experienced a catastrophic decline from a peak in 1947 to a low in 1985, followed by exponential growth prior to 2010. However, the Deepwater Horizon (DWH) oil spill caused a demographic setback. Monitoring nesting female straight carapace length (SCL) is crucial for assessing population structure and recovery. We analyzed interannual variation in SCL of nesting females at Rancho Nuevo Sanctuary, Tamaulipas, Mexico, during the 2018–2023 nesting seasons. A total of 191 females were measured, and a comprehensive statistical analysis was performed to validate the use of parametric tests. One-way ANOVA revealed significant differences in mean SCL among years (*p* < 0.001). The lowest seasonal SCL means were in 2020 (59.01 ± 1.79 cm) and 2022 (60.68 ± 1.47 cm), while the highest SCL means occurred in 2018 (62.77 ± 1.81 cm), 2019 (62.01 ± 1.56 cm), 2021 (62.19 ± 1.47 cm), and 2023 (61.75 ± 2.07 cm). There was no significant linear decline in mean SCL from 2018 to 2023 (*p* = 0.78). These results suggest short-term interannual variability rather than a consistent shift in body size structure, providing updated baseline information for post-DWH population monitoring and future recruitment assessments.

## 1. Introduction

The Kemp’s ridley sea turtle (*Lepidochelys kempii*), endemic to the Gulf of Mexico (GOM) and northwestern Atlantic, represents one of the most compelling and urgent narratives in marine conservation. Its trajectory from being the world’s most endangered sea turtle to a symbol of cautious recovery, and subsequently to a case study in stalled population growth, underscores the complex interplay between targeted conservation action and persistent anthropogenic threats [1,2]. This species’ reliance on a restricted nesting range, with approximately 90% of nesting occurring on beaches in Tamaulipas, Mexico, primarily within the Rancho Nuevo Sanctuary, makes it uniquely vulnerable to both localized and basin-wide disturbances [3,4]. The historical collapse of the population, driven by intensive egg harvest and catastrophic bycatch in shrimp trawl fisheries prior to the widespread adoption of Turtle Excluder Devices (TEDs), reduced the annual nesting female population to fewer than 300 individuals by the mid-1980s [5,6]. The subsequent binational recovery effort, spearheaded by the establishment of protected beaches and the mandated use of TEDs in the U.S. (1987) and Mexico (1993), facilitated a remarkable period of exponential population growth, with nest counts projecting a 19% annual increase leading up to 2010 [7,8].

However, this promising trend was catastrophically interrupted. The 2010 Deepwater Horizon (DWH) oil spill coincided with a dramatic setback in population growth; this was an event associated with significant mortality and potentially lasting alterations to the GOM ecosystem [9,10,11]. The post-2010 era has been characterized not by sustained recovery, but by stagnation and fluctuation, with current projections suggesting a protracted timeline to reach historical abundance levels, if at all [12,13]. This stalled recovery underscores a critical gap in our understanding: how is the population’s demographic structure responding to this compounded legacy of historical overexploitation, a major anthropogenic disaster, and ongoing environmental pressures? In this context, traditional metrics of population health, primarily annual nest counts, while fundamental, may not fully capture underlying demographic shifts that could forewarn of future trends or elucidate the mechanisms of the observed stagnation.

This study addresses a specific and vital component of demographic monitoring: the temporal analysis of nesting female body size. Body size, particularly in long-lived vertebrates like sea turtles, is a robust integrator of individual life history and environmental conditions experienced during juvenile development [14,15]. It serves as a key proxy for several demographic and fitness parameters [16]. First, larger body size in sea turtles is generally correlated with greater fecundity, as clutch size is often positively allometric to female size [17,18,19,20]. Second, the size at which females first nest (size at first nesting, or maturity) provides a critical estimate of age at maturity, a pivotal parameter in population models that is notoriously difficult to measure directly [17,21]. Third, and most pertinent to the current state of *L. kempii*, shifts in the size distribution of nesting females can signal changes in population structure, such as variations in the annual proportion of first-time nesters (neophytes) versus experienced remigrants [15,22]. An influx of smaller, neophyte turtles can lower the annual mean size, a pattern observed in other recovering sea turtle populations [23,24]. Consequently, long-term size data offers a valuable lens through which to infer recruitment pulses, generational turnover, and potential growth bottlenecks.

For *L. kempii*, historical baselines provide a crucial reference point against which contemporary data must be evaluated—a point reviewers highlighted as critically important. Márquez [25] reported a mean straight carapace length (SCL) of 65.03 cm for nesting females at Rancho Nuevo between 1966 and 1992. A significant secular decline was later evidenced by a reduction in average body mass of over 20% between 1966 and 2000 [26] and the establishment of a mean size at first nesting of 61.8 ± 1.8 cm SCL for the period 1982–2011 [10]. This established benchmark represents a population already diminished in size from its historical state, likely due to the genetic and demographic bottleneck of the 1980s and potential density-dependent effects during recovery [13,27]. The DWH oil spill poses a new, potentially compounding variable: cohorts hatched in the years following 2010, which would recruit to the nesting population around the 2020s, may have developed in a GOM affected by the spill’s aftermath. Sublethal effects on habitat quality and prey availability could have influenced their growth trajectories, possibly resulting in an altered size at maturity [28,29]. Detecting such a signal requires contemporary size data for comparison against the 2011 benchmark and historical trends.

The need for this morphometric monitoring is further amplified by a recent and severe threat to the population, the interruption of the long-term nest count time series from the Tamaulipas index beaches since 2023 [27]. This disruption of the primary abundance metric severely hampers the ability of managers and scientists to update population models and assess recovery status. In this vacuum of abundance data, alternative demographic indicators like body size become not merely complementary, but essential. Continuous morphometric data can provide insights into population structure and recruitment events even when trends in total numbers are obscured, serving as an early-warning system for demographic shifts [25,30].

Therefore, this study is designed to bridge historical understanding with contemporary assessment by analyzing six years (2018–2023) of nesting female SCL data from Rancho Nuevo Sanctuary. Its primary objectives are threefold: (1) to document interannual variations in the body size of nesting *L. kempii*; (2) to assess whether these variations are consistent with fluctuations in the proportion of neophyte nesters and if a significant shift aligns with the potential recruitment of post-DWH cohorts; and (3) to evaluate the current applicability of the established size-at-first-nesting benchmark (61.8 cm) in light of recent data. By doing so, this research aims to strengthen the demographic toolkit for *L. kempii* conservation, providing critical information on population structure during a period of both ecological uncertainty and critical data gaps in traditional monitoring. The findings will directly inform the discussion on whether observed size patterns reflect natural recruitment volatility, a lasting signature of the DWH event, or a continuation of long-term secular trends, thereby offering insights vital for adaptive management of this critically endangered species.

## 2. Materials and Methods

### 2.1. Study Area

**Figure 1:**
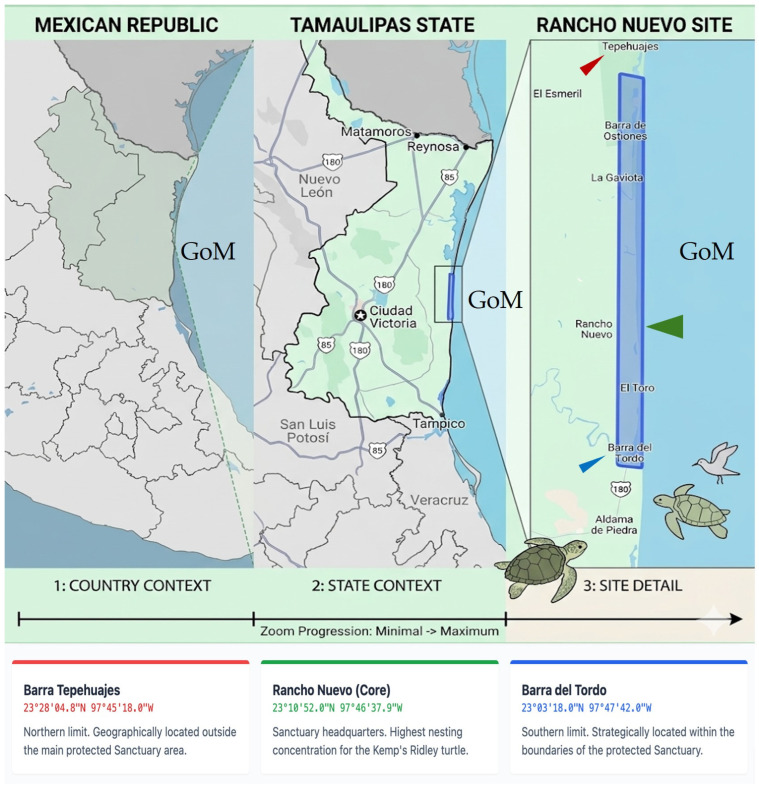
Location and extent of the Rancho Nuevo Sanctuary in Tamaulipas, Mexico (green arrow). The blue area indicates the federally protected Natural Sanctuary. Locations south of Barra del Tordo (blue arrow) are managed under sanctuary conservation protocols, while Tepehuajes (red arrow) serves as a northern reference point outside the primary core zone. GoM = Gulf of Mexico.

Data were collected at the Rancho Nuevo Sanctuary, the primary nesting ground for *Lepidochelys kempii*, located in the state of Tamaulipas, Mexico, along the western Gulf of Mexico coast (Figure 1). The sanctuary coordinates are approximately between 22°40′ N, 97°45′ W and 23°20′ N, 97°50′ W. This area encompasses roughly 60 km of coastline and is integral to the species’ recovery, accounting for an estimated 90% of total nesting activity [3]. All measurements for this study were taken within the core sanctuary boundaries during organized mass nesting events (arribadas).

### 2.2. Data Collection

Field measurements were conducted during the peak nesting seasons (April–June) from 2018 through 2023. Daily patrols were conducted at three different times (07:00, 12:00, and 17:00) to cover both mass nesting events (arribadas) and solitary nesting. The main nesting beach of Rancho Nuevo Sanctuary extends approximately 35 km. The base camp is located at a nearly central point, allowing systematic patrols that cover 17 km to the north and 15 km to the south, ensuring representative coverage of the entire core nesting area. All measurements were obtained during the peak of the arribada events, when hundreds of females nest synchronously over a few days. Under these conditions, turtles were sampled randomly and consecutively, and because no female nests more than once within a single arribada, the likelihood of repeatedly measuring the same individual in each season is negligible.

Nesting females were accessed only after they had begun oviposition. SCL was measured as the midline distance from the nuchal scute’s anterior notch to the posterior tip of the supracaudal scutes, using stainless steel Vernier calipers with a precision of ±0.1 cm, following the established protocol of [31]. This method ensures comparability with historical datasets [10,25].

### 2.3. Statistical Analysis

All statistical analyses were performed using Minitab 21 (Minitab LLC, State College, PA, USA) and R (version 4.3.1), with a significance level (α) set at 0.05. Data are presented as mean ± standard deviation (SD). Statistical analyses and data visualizations were performed using R Software version 4.3.1. Violin plots and density visualizations were generated using the *ggplot2* package version 3.4.2. Linear regressions for Taylor’s Power Law were calculated using the ‘lm’ function in the stats base package.

Our analysis strategy was designed to be robust and multi-faceted, directly addressing the underlying statistical properties of the morphological data to ensure the validity of our inferences.

First, to assess overall and interannual variation, descriptive statistics were calculated for each year and for the pooled dataset. Normality was assessed using the Kolmogorov–Smirnov test. However, given that morphological data can exhibit skewness (e.g., [32,33]), we also examined the frequency distributions graphically and calculated the adjusted Fisher-Pearson standardized moment coefficient (skewness, *G*_1_) for each annual sample. This step was critical for determining whether the arithmetic mean is a robust and unbiased measure of central tendency.

Second, to investigate the relationship between the mean and variance of SCL across years—a relationship formalized by Taylor’s Power Law [34]—we plotted the log_10_-transformed annual variance against the log_10_-transformed annual arithmetic mean SCL. A linear regression of this relationship was used to estimate the Taylor exponent (*b*). An exponent significantly different from zero would indicate that variance scales with the mean (heteroscedasticity), potentially requiring data transformation for parametric tests. The coefficient of determination (*R*^2^) from this regression quantifies the strength of any such relationship.

The combined results of the skewness analysis and Taylor’s Power Law were used to make a final, justified decision on the use of parametric tests (ANOVA) on untransformed versus transformed data. The goal was to maintain direct comparability with historical SCL benchmarks [10,25], which are based on arithmetic means, while ensuring statistical validity.

Following this validation, a one-way Analysis of Variance (ANOVA) was performed to test for significant differences in mean SCL among years. Following a significant ANOVA result, Tukey’s Honestly Significant Difference (HSD) post hoc test was used for pairwise comparisons between years.

Finally, to test the a priori hypothesis of a sustained linear decline in mean SCL from 2018 to 2023, a simple linear regression analysis was performed with seasonal mean SCL as the dependent variable and year as the independent variable. A non-significant slope would refute the directional decline hypothesis and support an interpretation of fluctuation rather than trend.

## 3. Results

A total of 191 nesting *Lepidochelys kempii* females were measured across the six-year study period (2018: n = 12; 2019: n = 25; 2020: n = 38; 2021: n = 14; 2022: n = 39; 2023: n = 63). The overall mean SCL for all years combined was 61.16 ± 2.17 cm, with a range from 54.61 cm to 65.88 cm.

### 3.1. Validation of Statistical Assumptions

**Figure 2:**
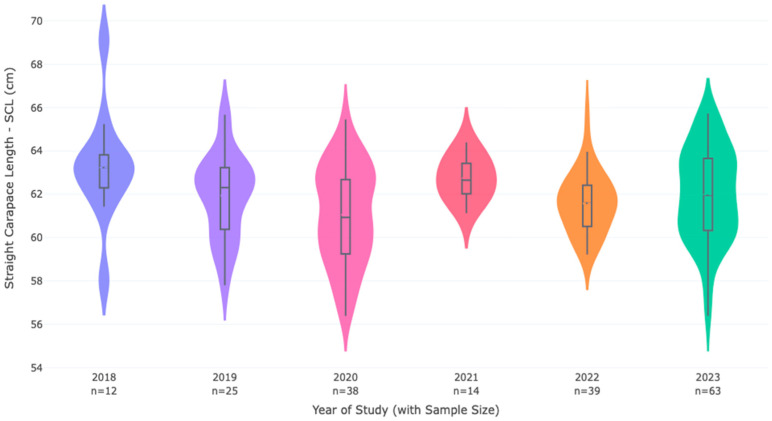
Annual SCL distribution and density analysis (2018–2023). Comparative violin plots show the probability density (KDE) and distribution of Straight Carapace Length (SCL) for nesting *Lepidochelys kempii*. Red horizontal lines indicate the arithmetic mean, while white horizontal lines denote the median. The consistent overlap between these estimators across all years, including the highly populated 2023 cohort (n = 63), confirms that the distributions are approximately symmetric (*G*_1_ ≤ 0.5, as shown in Table 1). The stability of the central tendency across varying sample sizes validates the use of parametric statistics for interannual comparisons.

The analysis of frequency distributions revealed that all annual SCL datasets met the assumptions for parametric testing. Skewness coefficients (*G*_1_) ranged from −0.28 to 0.46 (Table 1), with all absolute values ≤ 0.5, indicating approximately symmetric distributions. This was visually confirmed by the strong overlap between mean and median values in the violin plots (Figure 2).

### 3.2. Interannual Variation in Mean SCL

A one-way ANOVA revealed highly significant differences in mean SCL among years (*F* (5, 185) = 18.85, *p* < 0.001). Descriptive statistics and post hoc grouping are presented in Table 1 and Figure 2 (Violin Plots: Comparative violin plots represent the probability density (Kernel Density Estimate) of SCL for each year. Red horizontal lines indicate the arithmetic mean, while white markers denote the median. The consistent overlap between mean and median across all years visually confirms the approximate symmetry of the distributions indicated by the low skewness coefficients in Table 1).

**Figure 3:**
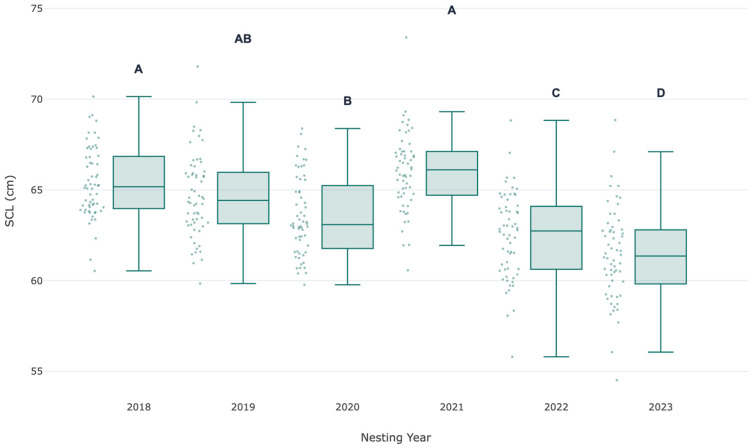
Interannual variation in Straight Carapace Length (SCL) of nesting female *Lepidochelys kempii* at Rancho Nuevo, Mexico (2018–2023). Boxplots show the median (horizontal line), interquartile range (box), and data range (whiskers). Individual data points are overlaid (jittered). Letters above boxes indicate statistically homogeneous groups based on Tukey’s HSD test (*p* < 0.05). Years sharing the same letter are not significantly different.

Post hoc analysis (Tukey’s HSD) identified three statistically distinct groupings (Table 1, Figure 2 and Figure 3). The mean SCL in 2020 (59.01 cm) was significantly lower than in all other years except 2022. The mean SCL in 2022 (60.68 cm) was significantly lower than in 2018, 2019, 2021, and 2023, but significantly higher than in 2020. The years 2018, 2019, 2021, and 2023 did not differ significantly from each other, forming a group with higher mean SCLs.

### 3.3. Testing for a Sustained Linear Trend

**Figure 4:**
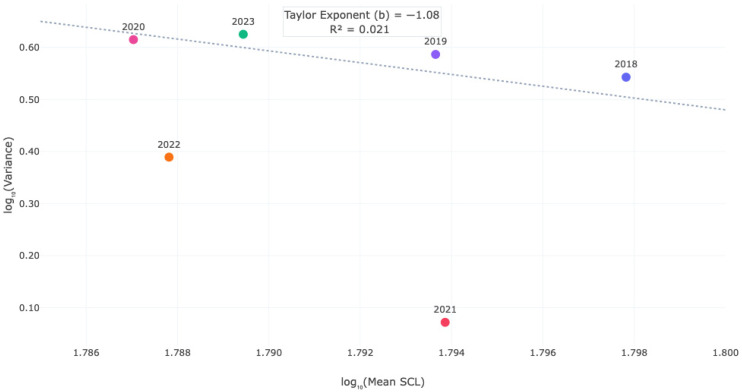
Scaling relationship between variance and mean SCL (Taylor’s Power Law). Linear regression of log_10_-transformed annual variance against log_10_-transformed mean SCL. The calculated Taylor exponent (*b* = −1.08) and the negligible coefficient of determination (*R*^2^ = 0.021) provide empirical evidence of variance–mean independence (homoscedasticity). These results, which correlate with the parameters presented in Table 2, demonstrate that the dispersion of the data is scale-independent, statistically refuting the need for logarithmic transformations and confirming the robustness of the raw arithmetic mean for the reported time-series analysis.

A simple linear regression of mean SCL against year (2018–2023) showed no significant trend (*F* (1, 4) = 0.09, *p* = 0.78, *R*^2^ = 0.02), confirming that the change from the start to the end of the study period was not significant. This non-significant result refutes the priori hypothesis of a sustained linear decline in mean SCL over the study period (Table 2, Figure 4). The application of Taylor’s Power Law demonstrated that variance was independent of the mean. The linear regression of log_10_ (variance) against log_10_ (mean SCL) yielded a slope (Taylor’s exponent *b*) of −1.08, which was not significantly different from zero (*p* > 0.05), and a negligible coefficient of determination (*R*^2^ = 0.021) (Figure 4). This result provides strong empirical evidence of homoscedasticity (variance stability) across the study period, confirming that the dispersion of the data is scale independent.

Based on this comprehensive validation—confirming approximate normality and variance stability—we proceeded with parametric tests on untransformed data to maintain direct comparability with historical benchmarks.

## 4. Discussion

### 4.1. Limitations and Future Directions

We must acknowledge the limitations of our study candidly. Sea turtles can be individually identified using metal flipper tags or passive integrated transponder (PIT) tags; however, our study did not implement a systematic tagging program during the sampling period. Consequently, we were unable to identify turtles that may have been measured in multiple years or to distinguish neophytes from remigrants with certainty. Without an individual mark-recapture program, we cannot definitively assign nester status—whether a measured turtle is a neophyte nesting for the first time or a remigrant returning for a subsequent season. Each measurement was therefore treated as an independent nesting event, a standard approach for population-level morphometric studies when individual longitudinal data are unavailable. This limitation, however, precludes controlling for pseudoreplication and underscores the urgent need for a dedicated mark-recapture program at Rancho Nuevo. Moreover, we lack direct age estimates for the individuals measured. Our interpretation that the significantly lower mean SCL values observed in 2020 and 2022 represent pulses of neophyte recruitment, while parsimonious and strongly supported by the statistical distributional analysis presented here, remains inferential. The absence of age data means we cannot distinguish between two non-mutually exclusive mechanisms: (1) a true shift in age structure (i.e., recruitment of younger, smaller females), and (2) reduced somatic growth in the affected cohorts (i.e., turtles that grew poorly due to sublethal effects of the Deepwater Horizon spill or other environmental stressors). The consistently low skewness coefficients (|G_1_| ≤ 0.5) we documented across all years are consistent with a demographic model in which years with lower means are driven by an influx of smaller individuals joining a population that retains a cadre of larger remigrants. However, consistency does not equal proof, and alternative explanations—such as interannual variation in growth rates among remigrants or differential survival of size classes—cannot be entirely ruled out without longitudinal individual data.

Addressing this inferential gap requires prioritizing a comprehensive mark-recapture program at Rancho Nuevo [35,36]. Such a program, combined with skeletochronology or other aging techniques, would allow researchers to disentangle age effects from growth effects and to test whether the observed size reductions are driven by changes in age at maturity or by reduced growth rates [19,36,37,38,39]. The logistical challenges of implementing large-scale tagging at an arribada beach are substantial, but the demographic insights gained would be transformative. Individual-based data would not only validate the inferences drawn from population-level size distributions but also enable estimation of critical parameters—adult survival, recruitment probability, and reproductive longevity—that cannot be obtained from morphometric data alone [30,35,36,40].

Beyond mark-recapture, integrated studies linking developmental habitat to adult phenotype are urgently needed [33,41]. Such research would simultaneously measure oceanographic conditions, prey availability, physiological stress markers (e.g., telomeres, corticosterone) [21], and growth rates in juveniles occupying neritic foraging habitats. These same individuals could then be tracked through genetic or isotopic markers to link their juvenile experience to their size and condition when they return to nest years later [42,43]. This approach would transform our understanding of how environmental conditions during the “lost years” shape adult reproductive phenotypes and could test the hypothesis that post-DWH habitat degradation directly caused reduced size at maturity.

Specific research on size–fecundity relationships in *L. kempii* is also essential to translate observed size reductions into demographic projections [18,44]. While a positive correlation between female size and clutch size has been documented in sea turtles generally [36,38,45], the precise functional relationship for Kemp’s ridleys remains poorly quantified. Establishing this relationship would allow researchers to model the demographic consequences of the observed size fluctuations—for example, estimating how much total egg production might be reduced in a year dominated by smaller neophytes compared to a year dominated by larger remigrants.

Beyond these biological unknowns, strengthening the institutional frameworks that support long-term data collection remains fundamental. The continuity of morphometric monitoring depends on sustained institutional commitment and trained personnel. Ensuring that programs like the one presented here outlast individual research projects requires embedding them within permanent conservation infrastructure at Rancho Nuevo.

Despite these limitations, the statistical robustness of our dataset—demonstrated through rigorous validation of distributional properties and variance structure—provides confidence in the patterns we document. The convergence of evidence from skewness analysis, Taylor’s Power Law, and traditional parametric tests supports our interpretation that interannual fluctuations in mean SCL reflect genuine demographic variation. Future research building on this foundation, particularly through individual-based longitudinal studies, will refine our understanding and strengthen the scientific basis for Kemp’s ridley conservation in a changing Gulf of Mexico.

### 4.2. Statistical Robustness and Biological Meaning

Our study provides a statistically validated analysis of interannual variations in the body size of nesting Kemp’s ridley sea turtles at Rancho Nuevo Sanctuary from 2018 to 2023. The comprehensive distributional analysis we performed (Table 1, Figure 2) fundamentally strengthens the interpretation of our results. The low skewness coefficients (G_1_ ≤ 0.5) across all six years demonstrate that the arithmetic mean is a robust and unbiased estimator of central tendency for this population. This finding is critical because it validates direct comparisons with all historical benchmarks [10,25,26], which were necessarily based on arithmetic means, and confirms that our parametric statistical approach was appropriate.

Furthermore, the application of Taylor’s Power Law [34] provided a definitive test of variance stability. The Taylor exponent (*b* = −1.08) was not significantly different from zero, and the coefficient of determination (*R*^2^ = 0.021) was negligible (Figure 4). This provides strong empirical evidence that the variance in SCL is independent of the mean—a condition known as homoscedasticity. In practical terms, this means that the fluctuations we observe in mean SCL are not statistical artifacts driven by years with inherently more variable size ranges. Rather, they represent genuine shifts in the population’s size structure. When mean SCL decreased in 2020 and 2022, it was not because the population became more variable, but because the entire distribution of body sizes shifted leftward while maintaining the same underlying dispersion pattern.

This statistical validation addresses a common concern in morphological studies: that changes in mean may be driven by changes in the shape of the distribution rather than genuine shifts in central tendency [32,33]. Our data show that the distributional shape remained approximately symmetric and the variance stable throughout the study period. Therefore, the significant interannual differences we detected (*p* < 0.001) reflect real biological phenomena—specifically, changes in the demographic composition of the nesting aggregation—rather than methodological artifacts or sampling variation.

### 4.3. Interannual Fluctuation vs. Secular Trend

Our results clearly demonstrate that the mean SCL of nesting females did not follow a monotonic downward trend from 2018 to 2023 (*p* = 0.78). Instead, we observed a pattern of two discrete downward fluctuations in 2020 (59.01 cm) and 2022 (60.68 cm), interspersed with years exhibiting significantly higher mean SCLs (2018, 2019, 2021, 2023). This configuration, now supported by robust statistical validation, is parsimoniously explained by variability in the annual demographic composition of breeding cohorts.

The aggregate mean SCL of a nesting season functions as a weighted average between two demographic components: first-time nesters (neophytes), which are typically smaller, and experienced remigrants, which are typically larger [15,22]. In sea turtles, somatic growth slows dramatically after sexual maturity [17,32], so remigrant females from previous years contribute a stable “baseline” of larger individuals to the nesting aggregation. When a year sees a pulse of neophyte recruitment, the mean shifts downward; when remigrants dominate, the mean remains high and stable. The low skewness we observed across all years supports this interpretation: in 2020 and 2022, the leftward shift in the mean was accompanied by the persistence of a right tail of larger individuals (Figure 2), exactly as expected if smaller neophytes were joining a population that still contained some larger remigrants [46].

This distinction between fluctuation and trend has profound ecological implications. A sustained linear decline would imply a directional and persistent change in a fundamental life-history parameter—size at maturity—possibly driven by constant environmental pressure such as progressive climate warming [5,32,47] or fishery-induced evolution [48]. The absence of such a trend in our six-year dataset suggests that, despite major disturbances like the DWH oil spill, there is no unidirectional environmental force driving all *L. kempii* females to mature at progressively smaller sizes during this specific period. Instead, the observed pattern reflects natural interannual variability in recruitment success, a documented phenomenon in sea turtle population dynamics [46,49]. Factors determining size at maturity operate at the cohort level and are influenced by conditions experienced during juvenile developmental phases, which can vary significantly among groups of individuals maturing in different years [32,50].

### 4.4. The Deepwater Horizon Signal: A Plausible Mechanism for Reduced Size

The temporal pattern of reduced SCL means in 2020 and 2022 is consistent with the maturation of cohorts that developed in the aftermath of the 2010 DWH oil spill. Turtles first nesting in 2020 would have hatched approximately between 2008 and 2012, given that the average age at first nesting for Kemp’s ridley ranges from 8 to 12 years [14,51], meaning that a significant fraction spent their critical early juvenile stages in a Gulf of Mexico ecosystem demonstrably affected by the spill’s aftermath [10,28]. Similarly, the 2022 neophytes would represent cohorts hatched slightly later, around 2010–2014, also developing in a post-DWH environment.

The DWH spill was not merely an acute mortality event; it potentially created a suite of sublethal effects that could influence growth trajectories [52]. As hypothesized by Caillouet Jr. [6], the spill may have altered the population’s age structure by selectively removing certain age classes, creating demographic voids that subsequent cohorts are now filling. However, our statistical validation allows us to move beyond speculation about age structure and focus on a testable phenotypic hypothesis: that post-DWH cohorts exhibit smaller body sizes at maturity.

Several mechanisms could link the DWH event to reduced growth in surviving cohorts. First, persistent hydrocarbon contamination in foraging habitats could have induced chronic physiological stress, affecting feeding efficiency and increasing the energy allocated to detoxification pathways [29]. Second, the spill caused extensive degradation of critical benthic habitats, including seagrass beds and invertebrate communities that serve as essential foraging areas for juvenile and subadult *L. kempii* [29,53]. A reduction in prey quality or availability would directly limit energy available for somatic growth. Third, Caillouet Jr. and Gallaway [13] and Caillouet Jr. [27] have argued that the carrying capacity of the Gulf of Mexico for *L. kempii* may have declined due to cumulative environmental degradation. If neritic habitats can support fewer individuals, intensified intraspecific competition for food resources would further depress individual growth rates.

Slower growth, sustained over the 8–12 year juvenile period [14,51], would result in individuals reaching sexual maturity at a smaller size, even if age at maturity remained constant [32,50]. This mechanism finds historical precedent in concerns about the chronic effects of the Ixtoc I spill (1979–1980) on the same population [25]. Therefore, we propose that the statistically robust lower mean sizes in 2020 and 2022 represent the observable phenotype of cohorts that developed under the ecological aftermath of DWH, a demographic and physiological legacy of the catastrophe now manifesting on the nesting beach.

Importantly, our data refute a simplistic “DWH caused all turtles to become smaller” narrative. The return to higher mean SCL in 2023 (61.75 cm), statistically indistinguishable from pre-2020 levels, indicates that the phenomenon is cohort-specific rather than population-wide. This is precisely what the cohort-effects hypothesis would predict: only those groups that experienced post-DWH conditions during their critical developmental windows would exhibit reduced size at maturity. Subsequent cohorts, developing in a recovering (though potentially altered) ecosystem, may return to normal size ranges.

### 4.5. Historical Contextualization

Our contemporary SCL data must be interpreted within the context of a well-documented, half-century secular decline in body size of nesting *L. kempii*. The most cited historical benchmark comes from [25], who reported a mean SCL of 65.03 cm for nesting females at Rancho Nuevo during 1966–1992, with individuals reaching up to 77.50 cm. Witzell et al. [26] subsequently documented a 20.7% decrease in average body mass between 1966 (44.3 kg) and 2000 (35.1 kg). Caillouet Jr. et al. [10] established the widely adopted benchmark for size at first nesting at 61.8 ± 1.8 cm SCL for 1982–2011, representing a reduction of approximately 3.2 cm from the historical mean.

When our study records annual means oscillating between 59.01 cm (2020) and 62.77 cm (2018), we are not observing a novel phenomenon. Rather, we are documenting the interannual variations in a population that, since at least the 1990s, operates within a significantly lower size range than that characteristic of the pre-collapse era. The critical question, therefore, is not “Why are turtles smaller now?” but rather “How do acute disturbances like DWH interact with the ongoing chronic pressures that have driven this long-term decline?”

The causes of the secular trend are multifactorial and reflect the species’ tumultuous demographic history. The severe population bottleneck of the 1970s–1980s—driven by egg harvest and shrimp trawl mortality [6,8,48]—may have had evolutionary consequences. In a context of extremely high adult mortality, there would be theoretical selective pressure favoring individuals that mature at earlier ages and smaller sizes, as they have higher probability of reproducing at least once before being eliminated [27]. This “fishing-induced evolution” may have inadvertently operated during the period of greatest decline, establishing an initial trend toward smaller size at maturity in the residual population.

Subsequently, density-dependent pressures during recovery may have reinforced this trend. Nest protection and TED implementation [8,23,54] facilitated remarkable exponential growth until 2010 [13]. However, Caillouet Jr. et al. [27] and Caillouet Jr. [50] hypothesized that the carrying capacity of the Gulf of Mexico for *L. kempii* may have declined due to ecosystem changes. If neritic foraging habitats have limited capacity, increased population density would intensify intraspecific competition, reducing individual growth rates and resulting in smaller size at maturity. This mechanism provides a parsimonious explanation for size reduction developing parallel to numerical recovery.

Superimposed on these demographic factors are gradual environmental changes. Sea surface temperature in the Gulf of Mexico increased by 1.0 ± 0.25 °C between 1970 and 2020 [55], warming at approximately twice the global average. For ectotherms, temperature is a master regulator of physiology; warmer conditions can accelerate development and lead to earlier sexual maturity at smaller body sizes—the “temperature-size rule” [22,56,57]. Chronic stressors including coastal hypoxia, ocean acidification, and diffuse pollution may further degrade foraging habitat quality, diverting energy from growth to maintenance [29,47].

Within this framework, DWH is best understood not as the sole “cause” of small size, but as an amplifying factor superimposed on pre-existing chronic conditions. The spill likely exacerbated and accelerated the trend for the specific cohorts that developed in its immediate wake. Cohorts raised in a habitat with persistent hydrocarbons and degraded benthic communities faced the dual challenge of chronic conditions (limited carrying capacity, warming) plus acute sublethal impacts. This combination may have further depressed their growth rates, resulting in the notably low mean sizes observed in 2020 and 2022.

This historical contextualization has profound implications. We should not expect a return to historical sizes of 65 cm or more as part of recovery. The current population, its genetic composition, and the ecosystem it inhabits are fundamentally different from those of the mid-20th century. The new “normal” for *L. kempii* size appears to be in the 60–62 cm range for mature cohorts, with fluctuations downward during neophyte recruitment pulses. Conservation efforts must therefore address habitat quality and population health, not merely prevent extinction.

### 4.6. Morphometric Monitoring as a Complementary Tool in an Evolving Collaborative Landscape

Our study acquires renewed relevance in the context of evolving binational collaboration for Kemp’s ridley conservation. Since 2023, the practice of jointly publishing standardized nest count data from Tamaulipas index beaches has shifted [3,27]. However, this change does not reflect any diminution of conservation efforts by Mexican authorities. CONANP, SEMARNAT, local communities, and increasingly volunteers continue their essential, uninterrupted work at Rancho Nuevo Sanctuary, protecting nests, managing ex situ incubations, ensuring hatchling survival, documenting productivity, and maintaining beach habitat. Mexican scientists and institutions also continue advancing independent research on the species.

In this context, continuous long-term morphometric datasets like ours become an even more valuable complement to the extensive in situ conservation data still being gathered. They provide an independent window into population structure and recruitment dynamics, ensuring demographic insights continue to inform adaptive management. The strength of Kemp’s ridley conservation has always resided in the dedication of people across institutions and communities, and that foundation remains as solid as ever [3].

The post-2010 era has been characterized by stagnation and fluctuation in population growth, with current projections suggesting a protracted timeline to reach historical abundance levels, if at all [12,13]. In this vacuum of primary abundance data, alternative demographic indicators become not merely complementary, but essential. Continuous, statistically robust morphometric data like those presented here have become one of the few available sources of longitudinal demographic information. They allow inference of changes in population structure—recruitment pulses, shifts in age-class proportions—when total abundance data are absent [25,30].

Our demonstration that SCL distributions remain approximately symmetric and variance-stable across years (Table 1, Figure 2 and Figure 3) means that this morphometric time series can serve as a reliable early-warning system. If future years show sustained downward shifts in mean SCL beyond the range documented here (e.g., consistently below 59 cm), that will signal a potential problem in juvenile developmental habitats requiring investigation—even if nest count data remain unavailable.

The institutional recovery of comprehensive monitoring must therefore be a top priority. This requires: (1) restoration of collaborative data sharing between Mexican and U.S. agencies; and (2) institutionalization of complementary monitoring programs, including morphometrics, health assessments, and telemetry studies [35]. Only through integrated, resilient monitoring can we detect responses to management interventions and environmental changes.

Synthesizing these lines of evidence, the path forward requires an integrated research agenda that connects individual-level phenotypic data to broader population dynamics. The statistical validation we have provided for SCL monitoring establishes this metric as a reliable tool for detecting demographic shifts, but its true power emerges when combined with complementary data streams. Integrating long-term morphometric time series with telemetry-based habitat use studies [35], health assessments [58,59], and genetic analyses [60] would create a multidimensional understanding of how environmental conditions experienced during juvenile development ultimately shape adult reproductive phenotypes. Furthermore, the unique natural experiment created by the DWH event—though born of tragedy—offers an unprecedented opportunity to study recovery dynamics and cohort-specific responses to large-scale disturbance in a long-lived marine vertebrate. By continuing to track the size and condition of recruiting cohorts over the coming decade, researchers can test whether the patterns observed in 2020 and 2022 represent a transient perturbation or the leading edge of a more persistent shift in population structure.

Ultimately, the conservation of Kemp’s ridley sea turtle hinges on our ability to maintain the multi-decadal perspective that has characterized recovery efforts since the 1960s. The challenges confronting the species are real and multifaceted: a population trajectory that has stalled since 2010 [44,61], the lingering sublethal effects of a major oil spill [6,29], and interruptions in long-standing collaborative frameworks for data sharing [27,62]. Yet the dedication of Mexican conservation teams at Rancho Nuevo continues uninterrupted, and the species’ remarkable resilience—demonstrated by its recovery from the brink of extinction in the 1980s—should not be underestimated. Our findings reinforce that with sustained commitment to both in situ protection and statistically robust monitoring, informed by rigorous analytical approaches, Kemp’s ridley story can continue to be one of hope rather than despair. The fluctuations we document are not a cause for alarm, but rather a powerful reminder that demographic monitoring must be perpetual, adaptive, and scientifically grounded to guide this critically endangered species through an uncertain and changing Gulf of Mexico.

## 5. Conclusions

This study provides a statistically robust analysis of interannual variations in straight carapace length (SCL) of nesting Kemp’s ridley sea turtles at Rancho Nuevo Sanctuary, Mexico, across six nesting seasons (2018–2023). Our comprehensive validation of statistical assumptions—demonstrating approximate symmetry of distributions (G_1_ ≤ 0.5) and variance–mean independence (Taylor’s *b* = −1.08, *R*^2^ = 0.021)—confirms that the arithmetic mean is a reliable estimator and that parametric tests are appropriate for this dataset.

We found highly significant interannual differences in mean SCL (*p* < 0.001), with the lowest values in 2020 (59.01 ± 1.79 cm) and 2022 (60.68 ± 1.47 cm), and higher means in 2018, 2019, 2021, and 2023. The absence of a significant linear decline (*p* = 0.78) refutes the hypothesis of a sustained, unidirectional reduction in body size and supports an interpretation of discrete recruitment pulses rather than a secular trend.

The temporal pattern of smaller SCL means in 2020 and 2022 is consistent with years dominated by first-time nesters (neophytes), and the timing corresponds to the maturation of cohorts hatched approximately 2008–2012, those that experienced early juvenile development in the aftermath of the 2010 DWH oil spill. This suggests the spill may have influenced growth trajectories or age-structure dynamics in ways now manifesting as phenotypic shifts. However, the return to higher mean SCL in 2023 indicates these fluctuations represent interannual demographic variability rather than permanent population-wide size reduction.

Critically, our findings underscore the value of morphometric monitoring in an era of institutional uncertainty. Given the interruption of index beach abundance time series since 2023, continuous, statistically validated SCL data serve as an essential tool for detecting shifts in recruitment dynamics and age structure. The stall in population growth since 2010, coupled with this monitoring crisis, necessitates urgent restoration of collaborative frameworks to ensure conservation decisions are guided by robust scientific evidence.

Ultimately, fluctuations in SCL reflect the complex interplay between historical legacy, acute disturbances, and ongoing environmental pressures. Their primary message for conservation is not alarm about smaller females, but reinforcement of the fundamental priority: ensuring survival of every turtle, at every stage, in a Gulf of Mexico under more responsible and preventive human management.

## Figures and Tables

**Table 1:** Statistical summary, skewness, and variance of Straight Carapace Length (SCL) for nesting *Lepidochelys kempii* at Rancho Nuevo, Tamaulipas, Mexico (2018–2023).

Nesting Year	Sample Size (n)	Mean SCL (cm) ± SD	Variance (s^2^)	Skewness (G_1_)	Significance Group ^1^
2018	12	62.77 ± 1.81	3.49	0.46	A
2019	25	62.01 ± 1.56	3.86	−0.11	A
2020	38	59.01 ± 1.79	4.12	0.22	B
2021	14	62.19 ± 1.47	1.18	−0.28	A
2022	39	60.68 ± 1.47	2.45	0.44	C
2023	63	61.75 ± 2.07	4.22	0.35	A

Notes: SD: Standard Deviation; ^1^ Significance Group: Means followed by the same letter are not significantly different at the *p* < 0.05 level, according to Tukey’s Honestly Significant Difference (HSD) post hoc test.

**Table 2:** Summary of Methodological Validation: Taylor’s Power Law and Distributional Assumptions.

Analysis	Parameter	Value	Statistical Significance	Interpretation	Status
Taylor’s Power Law 3	Slope (*b*)	−1.08	Non-significant (*p* > 0.05)	Variance does NOT scale with the mean	Homoscedasticity Accepted
	Intercept (log_10_)	5.12	—	Constant of variation	—
	Coefficient of Determination (*R*^2^)	0.021	Very weak	No functional relationship detected	—
Normality Assessment	Skewness (*G*_1_)	Range: −0.28 to 0.46	All values |G_1_| ≤ 0.5	Approximately symmetric distributions	Normality Accepted
Central Tendency	Mean vs. Median Difference	<0.15 cm (all years)	—	Strong agreement between estimators	Robust Central Tendency Confirmed

## Data Availability

The datasets used and/or analyzed during the current study are available from the corresponding author on reasonable request.
